# Endurance exercise prevents high-fat-diet induced heart and mobility premature aging and *dsir*2 expression decline in aging *Drosophila*

**DOI:** 10.18632/oncotarget.23292

**Published:** 2017-12-15

**Authors:** Deng-Tai Wen, Lan Zheng, Fan Yang, Han-Zhe Li, Wen-Qi Hou

**Affiliations:** ^1^ Key Laboratory Of Physical Fitness and Exercise Rehabilitation of Hunan Province, Hunan Normal University, Chang Sha, 410012, Hunan Province, China

**Keywords:** premature aging, high-fat-diet, exercise, dSir2, cardiac function, Gerotarget

## Abstract

High-Fat-Diet (HFD)-induced obesity is a major contributor to heart and mobility premature aging and mortality in both *Drosophila* and humans. The *dSir*2 genes are closely related to aging, but there are few directed reports showing that whether HFD could inhibit the expression *dSir*2 genes. Endurance exercise can prevent fat accumulation and reverse HFD-induced cardiac dysfunction. Endurance also delays age-relate functional decline. It is unclear whether lifetime endurance exercise can combat lifetime HFD-induced heart and mobility premature aging, and relieve the harmful HFD-induced influence on the *dSir*2 gene and lifespan yet. In this study, flies are fed a HFD and trained from when they are 1 week old until they are 5 weeks old. Then, triacylglycerol levels, climbing index, cardiac function, lifespan, and *dSir*2 mRNA expressions are measured. We show that endurance exercise improves climbing capacity, cardiac contraction, and *dSir*2 expression, and it reduces body and heart triacylglycerol levels, heart fibrillation, and mortality in both HFD and aging flies. So, lifelong endurance exercise delays HFD-induced accelerated age-related locomotor impairment, cardiac dysfunction, death, and *dSir*2 expression decline, and prevents HFD-induced premature aging in *Drosophila*.

## INTRODUCTION

HFD-induced obesity contributes to locomotor impairment, lipotoxic cardiomyopathy, and lifespan decreases in both *Drosophila* and humans. Fly locomotor impairment declines with aging because of the weakened resistance to oxidative stress, and HFD-induced locomotor impairment is also closely associated with oxidative stress in *Drosophila* [1]. In humans, obesity is a chronic metabolic disease that has become a global problem. A lifestyle including an inappropriate diet (such as HFD) and exercise habits (sedentary habits), genetic factors, and an ‘obesogenic’ environment are the major contributing factors. Increasing evidence has confirmed that HFD-induced obesity is a well-established risk factor for most cardiovascular diseases, including coronary heart disease, heart failure, and atrial fibrillation, which is a serious issue in older people. Besides, lipid-rich diets and obesity are associated with worldwide ‘‘epidemics’’ of cardiovascular diseases, type 2 diabetes, cancer, and locomotor impairment [2–4]. Reports have indicated that a lipid-rich diet is associated with obesity and a reduced lifespan in both Drosophila and humans [5–7]. These studies suggested that HFD-induced obesity might be a major contributor to premature aging.

However, endurance exercise, an economical and non-invasive intervention, can efficiently prevent obesity and delay premature senescence. For example, from the perspective of energy metabolism, triacylglycerol(TAG), the major component in lipid storage, is essential for normal physiology. However, long-term excessive accumulation of TAG will very likely causes obesity in adipose tissue and is associated with organ dysfunction in nonadipose tissue [8]. Endurance exercise can consume triacylglycerol and supply energy to the body since skeletal muscle participates in the metabolism of fat, which suggests that exercise could reduce excessive accumulation and obesity in the body [9]. Exercise training causes positive changes in skeletal muscle capability and cardiac performance, and delays progressive declines in locomotor ability by strengthening skeletal muscle performance, thus reducing the incidence of several age-related events such as debilitating injuries due to falls [10, 11]. Moreover, endurance exercise benefits late-life retention of contractility and fractional shortening, and exercising individuals have a reduced incidence of fibrillation events [12–14]. A recent study reported that exercise training protects against age-dependent cardiac fibrosis by suppressing AT1R and Nox2 as part of a RAS-Nox2-TGF-beta pathway [15]. Increasing evidence suggests that exercise training does not only improve health-related-quality of life but also extends the average lifespan [16, 17]. These reports indicate that although exercise training has an opposite effect on the body compared with obesity, but there is no direct evidence that lifetime exercise training can totally combat lifetime HFD-induced obesity problems such as locomotor impairment, lipotoxic cardiomyopathy, and decrease in lifespan.

Accumulating evidence suggests that Sirt1/dSir2 play important roles not only in aging but also in lipid metabolism and obesity. On one hand, stimulation of Sirt1/dSir2 by overexpression of *Sirt1/dSir2*, sirtuin-activating compounds or calorie restriction is sufficient to induce a prolonged lifespan in yeast, *Caenorhabditis elegans*, *Drosophila melanogaster* and mice [18–21], and spontaneous aging is accompanied-by decreased expression of *Sirt1/dSir2* [22]. On the other hand, *Sirt1/dSir2* can regulate lipid metabolism and obesity via some target histones, transcription factors, co-regulators, and metabolic enzymes, such as peroxisome proliferator-activated receptor g (PPARg) coactivator-1 (PGC-1), which is a key regulator of thermogenesis in brown adipose tissue; and controls mitochondrial fatty acid oxidation. Furthermore, decreased cardiac *PGC-1a* expression is associated with cardiac lipid accumulation and subsequent heart failure [23–24]. Sir2 could regulate the activity of PGC-1 by its NAD-dependent deacetylation. These studies show that Sirt1 is the link between obesity and aging. Meanwhile, there is no evidence whether lifelong endurance exercise can relieve harmful HFD-induced influence on the *dSir2* gene and prevent premature aging.

Therefore, to study whether lifetime exercise could combat lifetime HFD-induced heart and mobility premature senescence and *dSir2* expression decline in flies, we took advantage of flies’ short lifespan. Flies were trained and fed HFD from when they were 1 week old until they were 5 weeks old. Then we measured the flies’ TAG levels, climbing index, cardiac function, lifespan, and *dSir2* mRNA expression. Based on these results, we tried to determine that whether exercise could prevent flies from HFD-induced premature aging and preliminarily analyze the possible mechanism behind this.

## RESULTS

### Exercise reduced HFD-induced and age-relate fat accumulation

Recent studies reported that 5 days of the HFD led to obesity phenotypes in flies, and it also caused an increase in TAG levels [25, 31]. However, endurance exercise could effectively prevent humans from HFD-induced obesity, and it contributes to mitochondrial enzyme activity in both mammal and Drosophila [9, 32]. To explore whether lifetime endurance exercise could prevent HFD-induced accelerated age-relate lipid accumulation, we assessed the relative TAG levels of the whole fly body at different ages.

**Figure 1:**
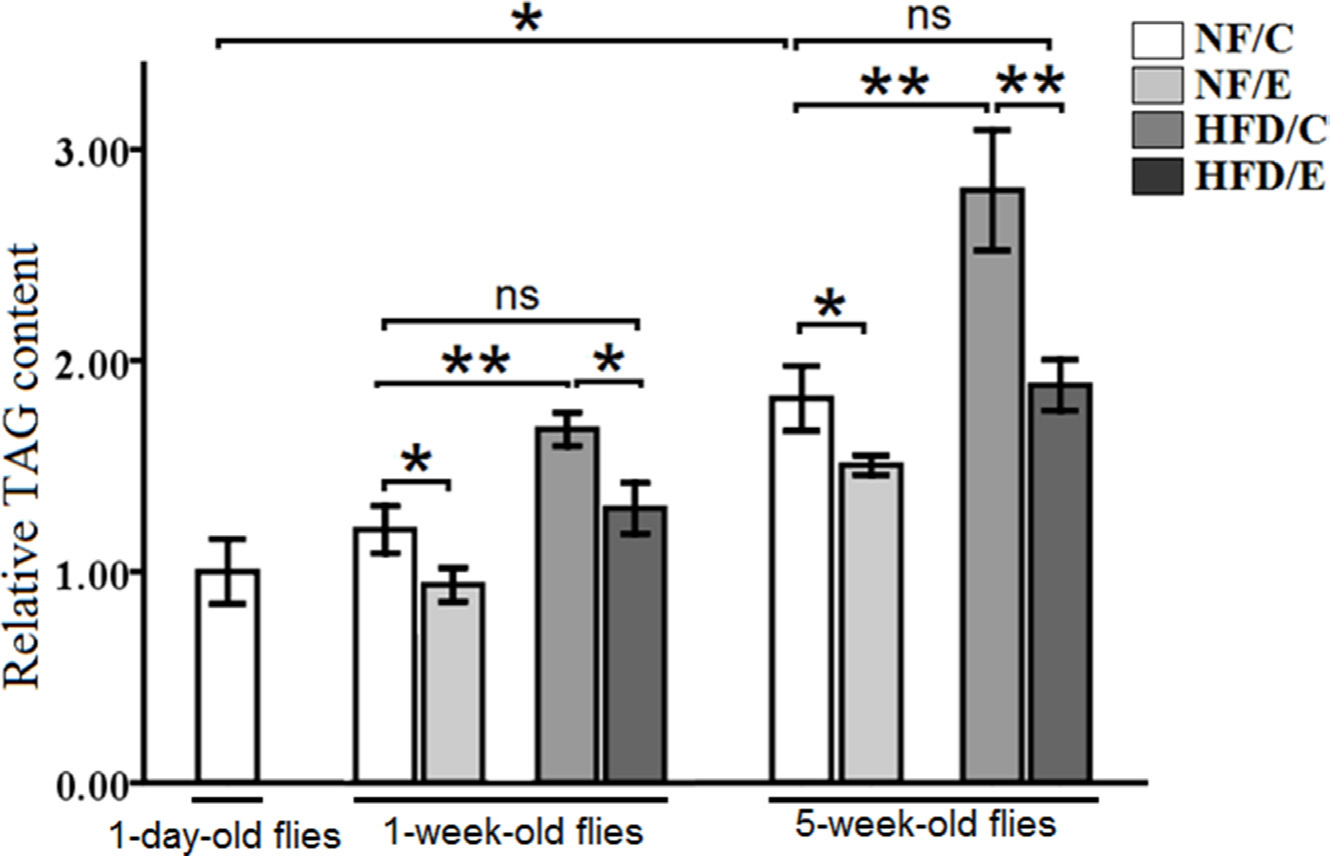
The relative TAG content in Drosophila The relative TAG levels were assessed at one-day old flies, one-week old flies, and five-week old flies. The relative TAG levels were normalized with fly weight. Independent-sample *t*-tests were used to assess differences in 1-day old and 5-week old in the NF/C group flies. Using a one-way analysis of variance (ANOVA) followed by an LSD test among the group NF/C, NF/E, HFD/C, and HFD/E. Sample size was 35 to 40 flies per group. Data are represented as means ± SEM. ^*^P < 0.05; ^**^P < 0.01.

We found that exercise significantly decreased TAG content (3-factor ANOVA, *P* < 0.01), HFD and age significantly increased TAG content (3-factor ANOVA, *P* < 0.01, *P* < 0.01); exercise and HFD had no interaction influence on TAG content (3-factor ANOVA, *P* > 0.05); exercise and age had no interaction influence on TAG content (3-factor ANOVA, *P* > 0.05); HFD and age had no interaction influence on TAG content (3-factor ANOVA, *P* > 0.05); exercise, HFD, and age had no interaction influence on TAG content (3-factor ANOVA, *P* > 0.05). We also found that the relative TAG content in 5-week-old flies was higher than in 1-day-old flies (Independent- sample *t*-tests, *P* < 0.05), which indicated that aging could induce a remarkable increase in TAG levels in Drosophila (Figure 1).

Besides, in 1-week-old flies, the relative TAG content in NF/E group flies was lower than NF/C group flies (LSD test, *P* < 0.05); the relative TAG content in HFD/C group flies was higher than NF/C group flies (LSD test, *P* < 0.01); the relative TAG content in HFD/E group flies was higher than HFD/C group flies (LSD test, *P* < 0.05), and there was no significant difference between NF/C group flies and HFD/C group flies in the relative TAG content(LSD test, *P* > 0.05) (Figure 1).

In 5-week-old flies, the relative TAG content in NF/E group flies was lower than NF/C group flies (LSD test, *P* < 0.05); the relative TAG content in HFD/C group flies was higher than NF/C group flies (LSD test, *P* < 0.01); the relative TAG content in HFD/E group flies was higher than HFD/C group flies (LSD test, *P* < 0.01), and there was no significant difference between NF/C group flies and HFD/C group flies in the relative TAG content(LSD test, *P* > 0.05) (Figure 1).

**Figure 2:**
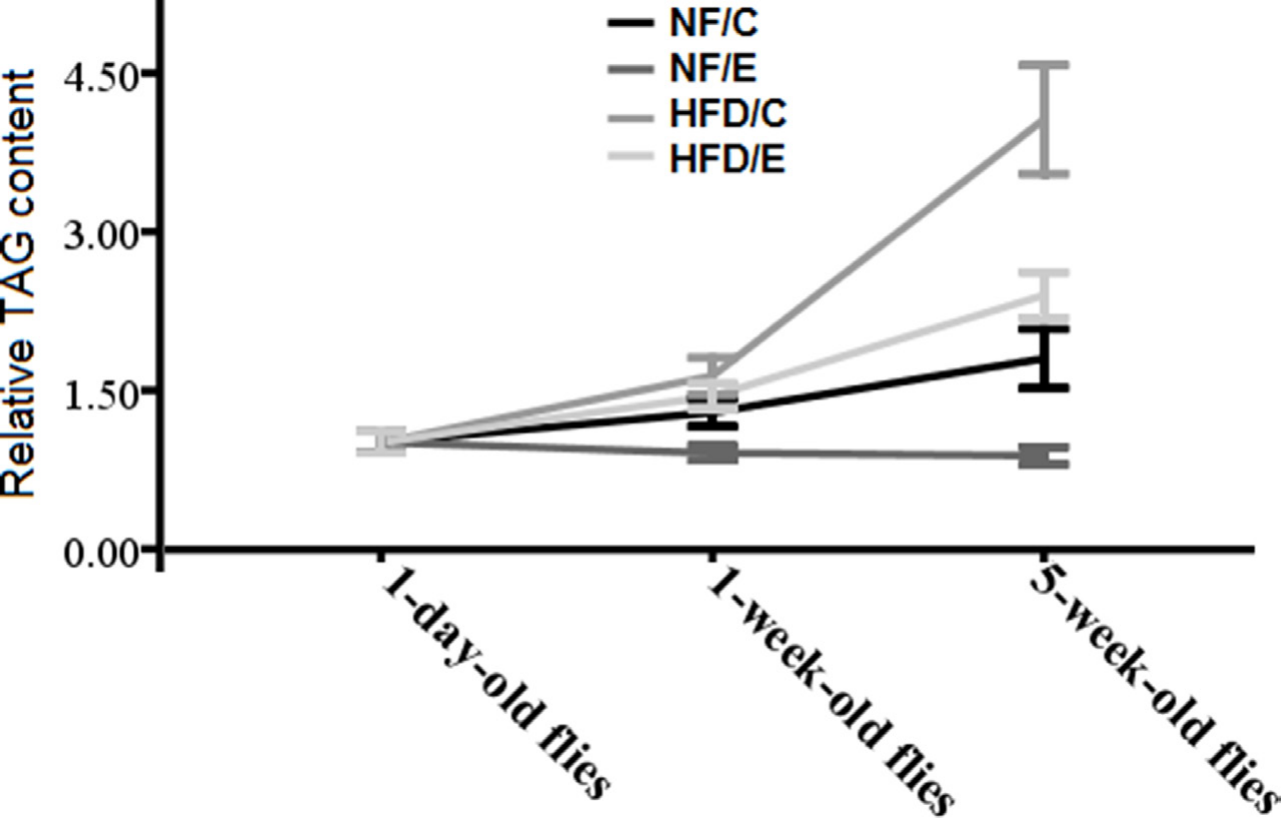
Effects of HFD and endurance training on flies’ relative TAG contents at different ages Results indicated that exercise could reduce flies’ age related fat accumulation, and HFD could badly accelerate flies’ age related fat accumulation. Exercise could slow HFD-induced age related fat accumulation.

**Figure 3:**
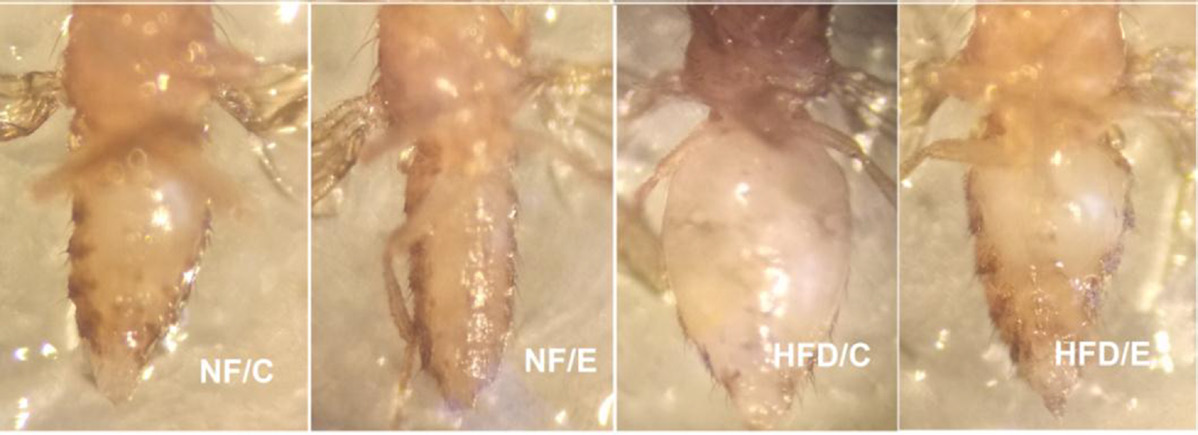
5-week old flies in different groups This picture clearly showed that HFD induced a higher body fat accumulation, but endurance exercise could reduce fat content and maintain lipids at a lower level.

These results suggested that HFD induced a higher body fat accumulation, while endurance exercise could reduce fat content and maintain lipids at a lower level in both young and old flies (Figure 1, Figure 2, and Figure 3 ).

### Exercise protected flies from HFD-induced and age-relate locomotor impairment

**Figure 4:**
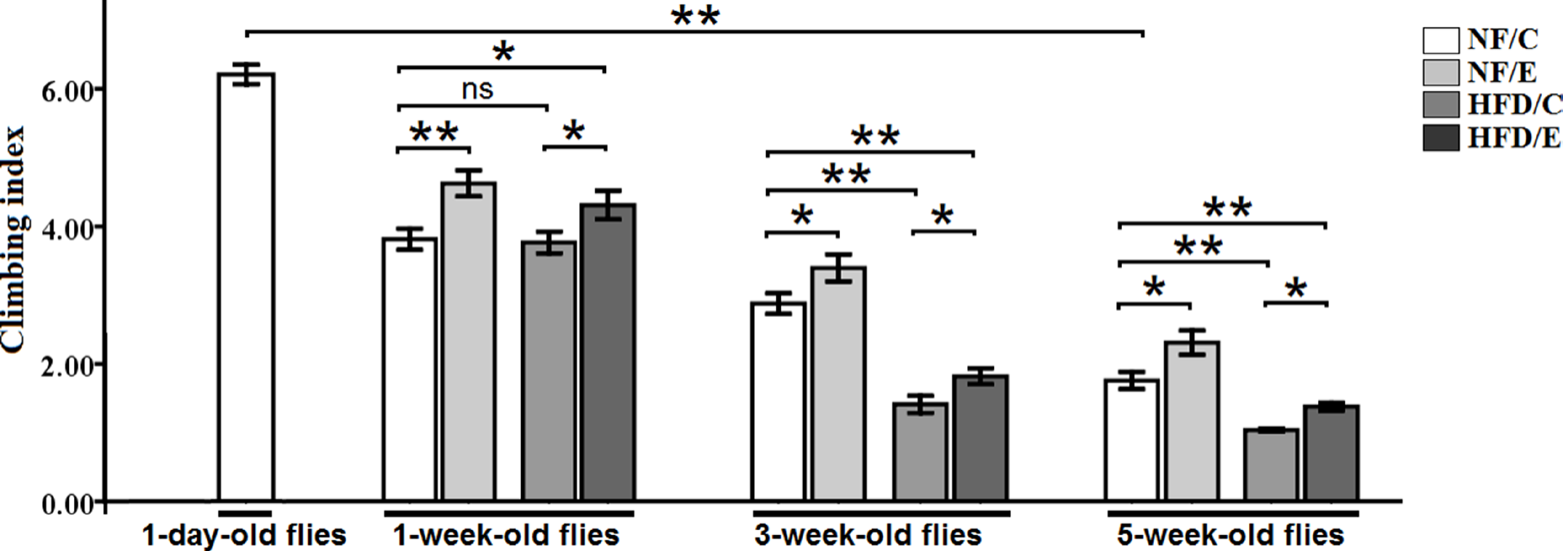
Effects of endurance training and HFD on locomotor impairment in *Drosophila* Flies average climbing index was assessed when flies were at 1-day-old flies, 1-week-old flies, 3-week-old flies, 5-week-old flies. Independent-sample *t*-tests were used to assess differences between 1-day-old flies and 5-week-old flies. Using a one-way analysis of variance (ANOVA) followed by an LSD test among the NF/C, NF/E, HFD/C, and HFD/E group flies. Sample size of climbing index was 100 to 110 flies per group. Data are represented as means ± SEM. ^*^P < 0.05; ^**^P < 0.01.

The climbing index reflected the capacity of negative geotaxis and activity, and was used as a general measure of mobility in flies [33]. In both humans and flies, locomotor impairment becomes serious with aging or obesity, and results in a health span and quality of life [1]. Exercise could improve mobility and maintain a good health span in both humans and flies through the gain of muscle mass and strength and by decreasing the levels of fat and connective tissue [11, 34, 35]; however, HFD-induced obesity could contribute to locomotor impairment [1]. There are few studies that report whether exercise could prevent HFD-induced and age related locomotor impairment [11, 34, 35]. To identify whether endurance exercise could improve HFD-induced and age-related lower mobility, we measured the impact of exercise and the HFD on the climbing index of flies at 1 week, 3 weeks, and 5 weeks age. We found that exercise significantly increased climbing index (3-factor ANOVA, *P* < 0.01), HFD and age significantly reduced climbing index (3-factor ANOVA, *P* < 0.01, *P* < 0.01); exercise and HFD had no interaction influence on the climbing index (3-factor ANOVA, *P* > 0.05); exercise and age had no interaction influence on the climbing index (3-factor ANOVA, *P* > 0.05); HFD and age had no interaction influence on the climbing index (3-factor ANOVA, *P* > 0.05); exercise, HFD, and age had no interaction influence on the climbing index (3-factor ANOVA, *P* > 0.05). We observed that the climbing index of 1-day-old flies was significantly higher than of the 5-week-old flies (independent-sample *t*-tests, *P* < 0.01)(Figure 4) , which suggested that flies’ locomotor impairment became worse with aging.

Besides, at the age of 1 week, the climbing index of NF/E group flies was significantly higher than of the NF/C group flies (LSD test, *P* < 0.01); The climbing index of HFD/E group flies was significantly higher than of the HFD/C group flies (LSD test, *P* < 0.05); The climbing index of HFD/E group flies was significantly higher than of the NF/C group flies (LSD test, *P* < 0.05) (Figure 4).

At 3 weeks old, the climbing index of NF/E group flies was significantly higher than of the NF/C group flies (LSD test, *P* < 0.05); The climbing index of HFD/C group flies was significantly lower than of the HFD/C group flies (LSD test, *P* < 0.01); The climbing index of HFD/E group flies was significantly higher than of the HFD/C group flies (LSD test, *P* < 0.05); The climbing index of HFD/E group flies was significantly lower than of the NF/C group flies (LSD test, *P* < 0.01) (Figure 4).

At 5 weeks old, the climbing index of NF/E group flies was significantly higher than of the NF/C group flies (LSD test, *P* < 0.05); The climbing index of HFD/C group flies was significantly lower than of the NF/C group flies (LSD test, *P* < 0.01); The climbing index of HFD/E group flies was significantly higher than of the HFD/C group flies (LSD test, *P* > 0.05); The climbing index of HFD/E group flies was significantly lower than of the NF/C group flies (LSD test, *P* < 0.05) (Figure 4).

These results indicated that exercise could protect young, adult, and old flies from HFD-induced locomotor impairment, which suggested that endurance exercise could delay HFD-induced accelerated age-relate locomotor impairment.

### Exercise protected the heart from HFD-induced cardiac TAG accumulation, heart rate increased, fraction shortening decreased, and fibrillation rise in flies

Lipotoxic cardiomyopathy, a form of cardiac dysfunction, is caused by excessive lipid accumulation in myocardial cells [36, 37]. Recent studies reported that HFD-induced obesity could lead to excessive fat accumulation accompanied by severe heart defects, including increased frequency of arrhythmias, reduced cardiac output, increased non-contractile myocardial cells, and altered myofibrillar structure and collagen content [38, 39]. However, accumulating evidence has reported that exercise can improve cardiac contractility and prevent heart diseases [12–14]. To identify whether endurance exercise could protect the heart from HFD-induced lipotoxic cardiomyopathy and age-related cardiac dysfunction, we measured the impacts of training and HFD on relative TAG level, heart rate, fraction shortening, and fibrillation.

**Figure 5:**
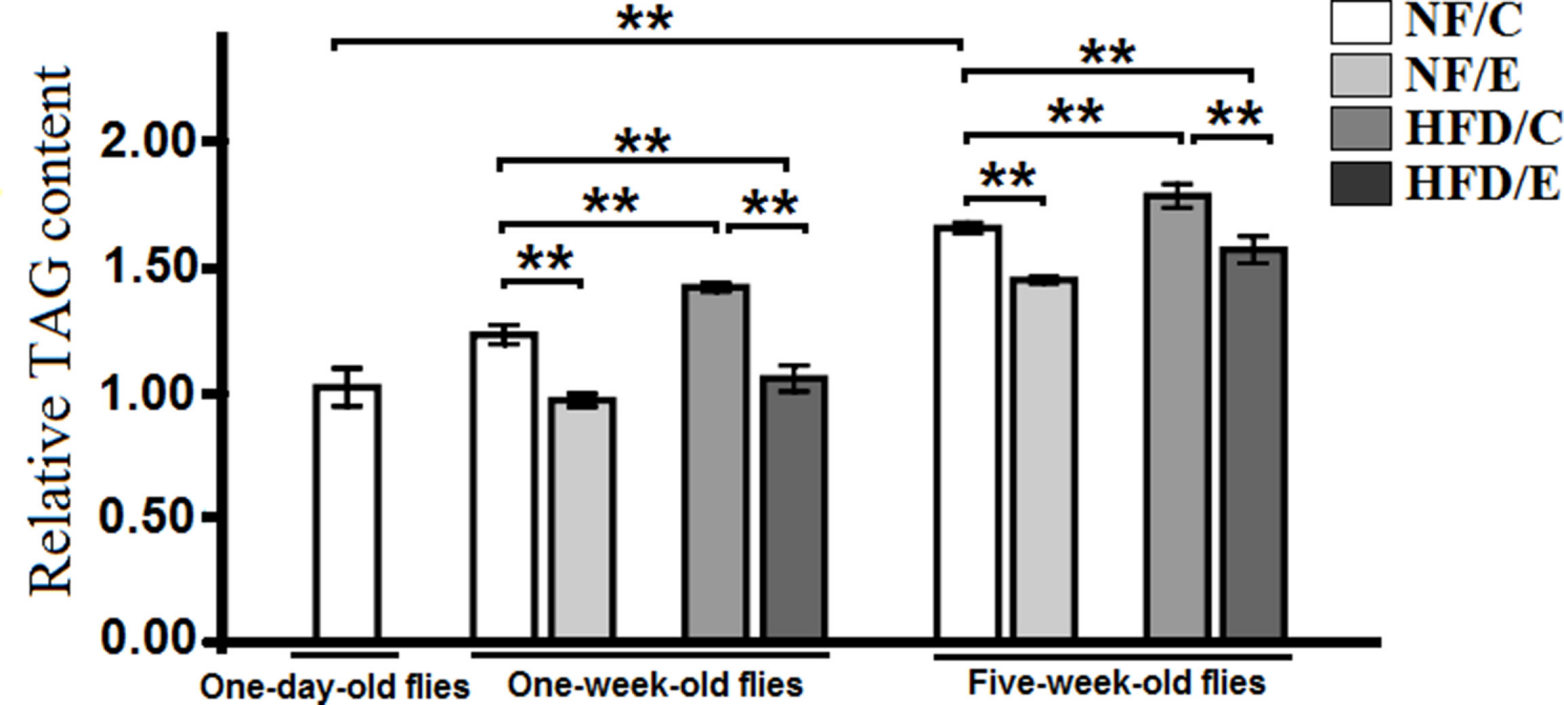
Effects of HFD and endurance training on the heart relative TAG level at one-day old flies, one-week old flies, and five-week old flies Independent -sample *t*-tests were used to assess differences between 1-day- old flies and 5-week-old flies. Using a one-way analysis of variance (ANOVA) followed by an LSD test among the NF/C, NF/E, HFD/C, and HFD/E group flies. Sample size of heart TAG content was 240 flies heart per group. Data are represented as means ± SEM. ^*^P < 0.05; ^**^P < 0.01.

**Figure 6:**
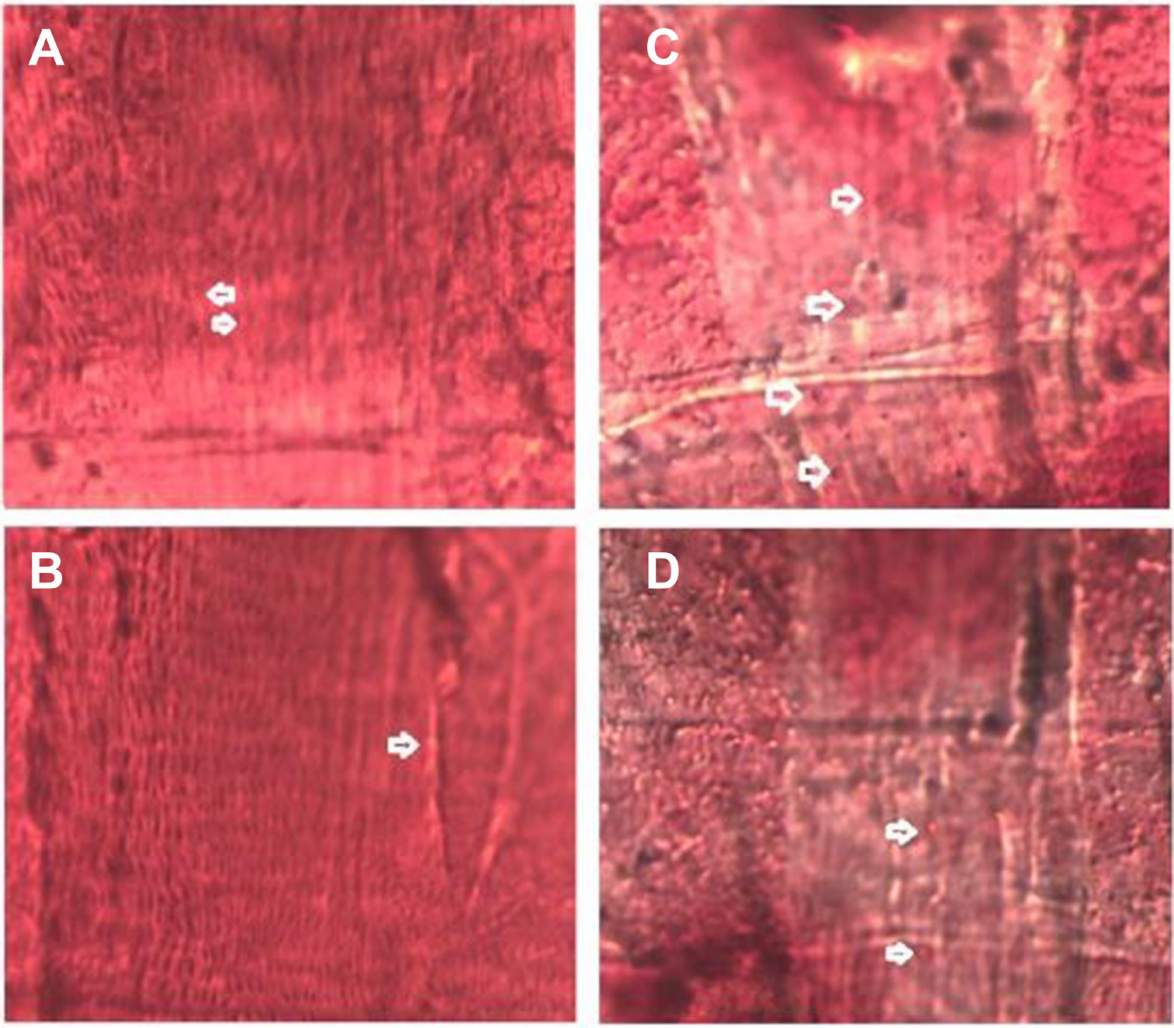
Oil Red O staining of cardiac muscle in five-week old flies (**A**) A few lipid droplets in NF/C group flies heart. (**B**) Very few lipid droplets in NF/E group flies heart. (**C**) A lot of lipid droplets in HFD/C group flies heart. (**D**) A few lipid droplets in HFD/E group flies heart.

In this study, we found that exercise significantly reduced TAG content (3-factor ANOVA, *P* < 0.01); HFD and age significantly inreased cardiac TAG content (3-factor ANOVA, *P* < 0.01, *P* < 0.01); exercise and HFD had no interaction influence on cardiac TAG content (3-factor ANOVA, *P* > 0.05); exercise and age had no interaction influence on cardiac TAG content (3-factor ANOVA, *P* > 0.05); HFD and age had no interaction influence on cardiac TAG content (3-factor ANOVA, *P* > 0.05); exercise, HFD, and age had no interaction influence on cardiac TAG content (3-factor ANOVA, *P* > 0.05). The heart relative TAG level of 1-day-old flies was significantly lower than of the 5-week-old flies (independent-sample *t*-tests, *P* < 0.01), which suggested that flies’ heart lipid accumulation became severe with aging. In 1 week old flies, the relative TAG level of NF/C group flies was significantly higher than of the NF/E group flies (LSD test, *P* < 0.01); the relative TAG level of HFD/C group flies was significantly higher than of the NF/C group flies (LSD test, *P* < 0.01); the hear relative TAG level of HFD/E group flies was significantly lower than of the NF/C group flies (LSD test, *P* < 0.01); the heart relative TAG level of HFD/E group flies was significantly lower than of the HFD/C group flies (LSD test, *P* < 0.05). At 5 weeks old, the heart relative TAG level of NF/E group flies was significantly lower than of the NF/C group flies (LSD test, *P* < 0.01); the heart relative TAG level of HFD/C group flies was significantly higher than of the NF/C group flies (LSD test, *P* < 0.01); the heart relative TAG level of HFD/E group flies was significantly lower than of the NF/C group flies (LSD test, *P* < 0.01); the heart relative TAG level of HFD/E group flies was significantly lower than of the HFD/C group flies (LSD test, *P* < 0.01)(Figure 5 and Figure 6).

**Figure 7:**
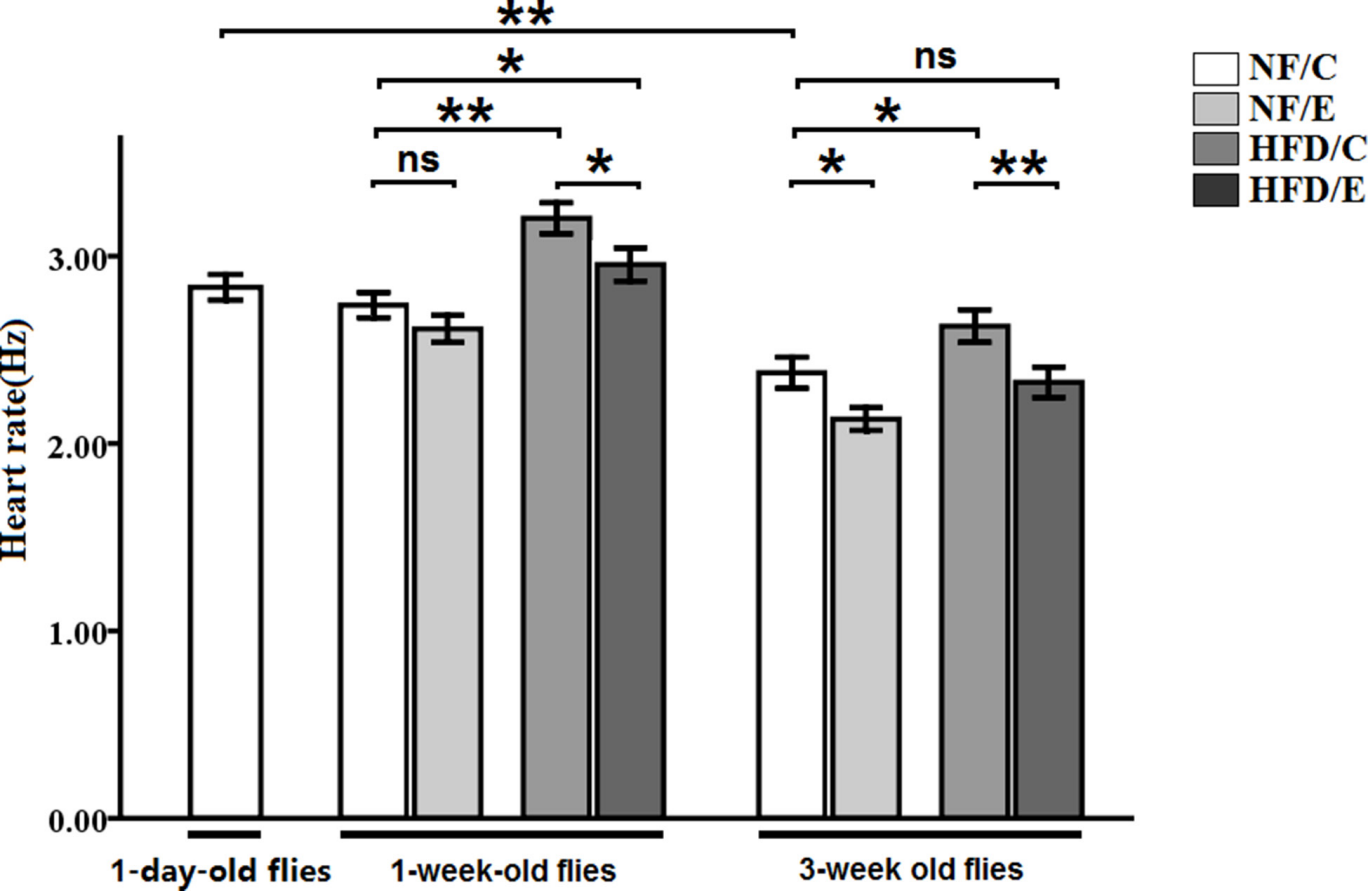
Effects of HFD and endurance training on heart rate at one-day old flies, one-week old flies, and five-week old flies Independent- sample *t*-tests were used to assess differences between 1-day-old flies and 5-week-old flies. Using a one-way analysis of variance (ANO VA) followed by an LSD test among the NF/C, NF/E, HFD/C, and HFD/E group flies. Sample size of heart rate was 30 flies per group. Data are represented as means ± SEM. ^*^P < 0.05; ^**^P < 0.01.

We found that exercise significantly reduced heart rate (3-factor ANOVA, *P* < 0.01); HFD and age significantly increased heart rate (3-factor ANOVA, *P* < 0.01, *P* < 0.01); exercise and HFD had no interaction influence on heart rate (3-factor ANOVA, *P* > 0.05); exercise and age had no interaction influence on heart rate (3-factor ANOVA, *P* > 0.05); HFD and age had no interaction influence on heart rate (3-factor ANOVA, *P* > 0.05); exercise, HFD, and age had no interaction influence on heart rate (3-factor ANOVA, *P* > 0.05). The heart rate of 1-day-old flies was significantly higher than of the 5-week-old flies (independent-sample *t*-tests, *P* < 0.01), which suggested that flies’ heart rate became slow with aging. In 1 week old flies, the heart rate of HFD/C group flies was significantly higher than of the NF/C group flies (LSD test, *P* < 0.01); the heart rate of HFD/E group flies was significantly higher than of the NF/C group flies (LSD test, *P* < 0.05); the heart rate of HFD/E group flies was significantly lower than of the HFD/C group flies (LSD test, *P* < 0.05). At 5 weeks old, the heart rate of NF/E group flies was significantly lower than of the NF/C group flies (LSD test, *P* < 0.05); the heart rate of HFD/C group flies was significantly higher than of the NF/C group flies (LSD test, *P* < 0.05); the heart rate of HFD/E group flies was significantly lower than of the HFD/C group flies (LSD test, *P* < 0.01) (Figure 7).

**Figure 8:**
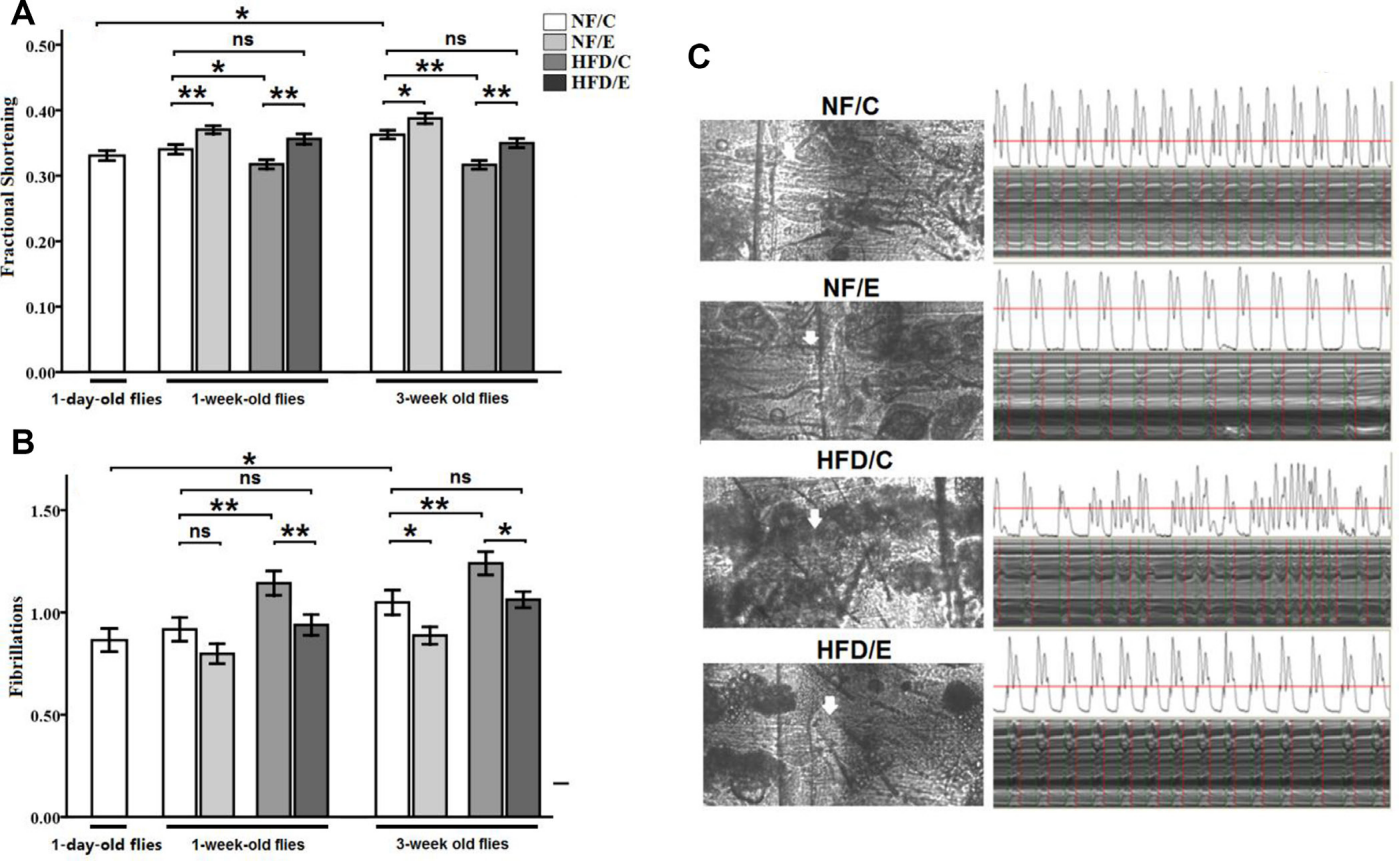
Effects of endurance training and HFD on fraction shortening and fibrillation at one-day old flies, one-week old flies, and five-week old flies (**A**) Fraction shortening. (**B**) Fibrillation. (**C**) Effects of HFD and endurance training on the fat storage in non-adipose tissue heart and percardial cells at five-week old. Heart and percardial cells were observed clearly in NF/C flies. Heart and percardial cells were also observed clearly in NF/E flies. Heart and percardial cells could not be observed clearly in HFD/C flies since there were much fat storage in non-adipose tissue heart and percardial cells after five-week HFD, and the heart would be damaged even though this fat was removed very carefully in order to see the heart tube clearly. Independent-sample *t*-tests were used to assess differences between 1-day-old flies and 5-week-old flies. Using a one-way analysis of variance (ANOVA) followed by an LSD test among the NF/C, NF/E, HFD/C, and HFD/E group flies. Sample size was 30 flies per group. Data are represented as means ± SEM. ^*^P < 0.05; ^**^P < 0.01.

Besides, we observed that exercise significantly improved fractional shortening (3-factor ANOVA, *P* < 0.01); HFD and age significantly reduced fractional shortening (3-factor ANOVA, *P* < 0.01, *P* < 0.01); exercise and HFD had no interaction influence on fractional shortening (3-factor ANOVA, *P* > 0.05); exercise and age had no interaction influence on fractional shortening (3-factor ANOVA, *P* > 0.05); HFD and age had no interaction influence on fractional shortening (3-factor ANOVA, *P* > 0.05); exercise, HFD, and age had no interaction influence on fractional shortening (3-factor ANOVA, *P* > 0.05). The fractional shortening of 1-day-old flies was significantly lower than of the 5-week-old flies (independent-sample *t*-tests, *P* < 0.01), which suggested that flies’ fractional shortening became increased with aging. In 1 week old flies, the fractional shortening of NF/E group flies was significantly higher than of the NF/C group flies (LSD test, *P* < 0.01); the fractional shortening of HFD/C group flies was significantly lower than of the NF/C group flies (LSD test, *P* < 0.05); the fractional shortening of HFD/E group flies was significantly higher than of the HFD/C group flies (LSD test, *P* < 0.01). At 5 weeks old, the fractional shortening of NF/E group flies was significantly higher than of the NF/C group flies (LSD test, *P* < 0.01); the fractional shortening of HFD/C group flies was significantly lower than of the NF/C group flies (LSD test, *P* < 0.05); the fractional shortening of HFD/E group flies was significantly higher than of the HFD/C group flies (LSD test, *P* < 0.01) (Figure 8A).

Finally, we observed that exercise significantly reduced fibrillation (3-factor ANOVA, *P* < 0.01); HFD and age significantly inreased on fibrillation (3-factor ANOVA, *P* < 0.01, *P* < 0.01); exercise and HFD had no interaction influence on fibrillation (3-factor ANOVA, *P* > 0.05); exercise and age had no interaction influence on fibrillation (3-factor ANOVA, *P* > 0.05); HFD and age had no interaction influence on fibrillation (3-factor ANOVA, *P* > 0.05); exercise, HFD, and age had no interaction influence on fibrillation (3-factor ANOVA, *P* > 0.05). The fibrillation of 1-day-old flies was significantly lower than of the 5-week-old flies (independent-sample *t*-tests, *P* < 0.05), which suggested that flies’ fibrillation became worse with aging. At 1 week old, the fibrillation of HFD/C group flies was significantly higher than of the NF/C group flies (LSD test, *P* < 0.01); the fibrillation of HFD/E group flies was significantly lower than of the HFD/C group flies (LSD test, *P* < 0.01). At 5 weeks old, the fibrillation of NF/E group flies was significantly lower than of the NF/C group flies (LSD test, *P* < 0.05); the fibrillation of HFD/C group flies was significantly higher than of the NF/C group flies (LSD test, *P* < 0.01); the fibrillation of HFD/E group flies was significantly lower than of the HFD/C group flies (LSD test, *P* < 0.05) (Figure 8B and 8C).

These results suggested that exercise could reduce lipid droplets in heart, and protect heart from HFD-induced damage at different ages.

### Exercise protected flies from HFD-induced lifespan reduction

**Figure 9:**
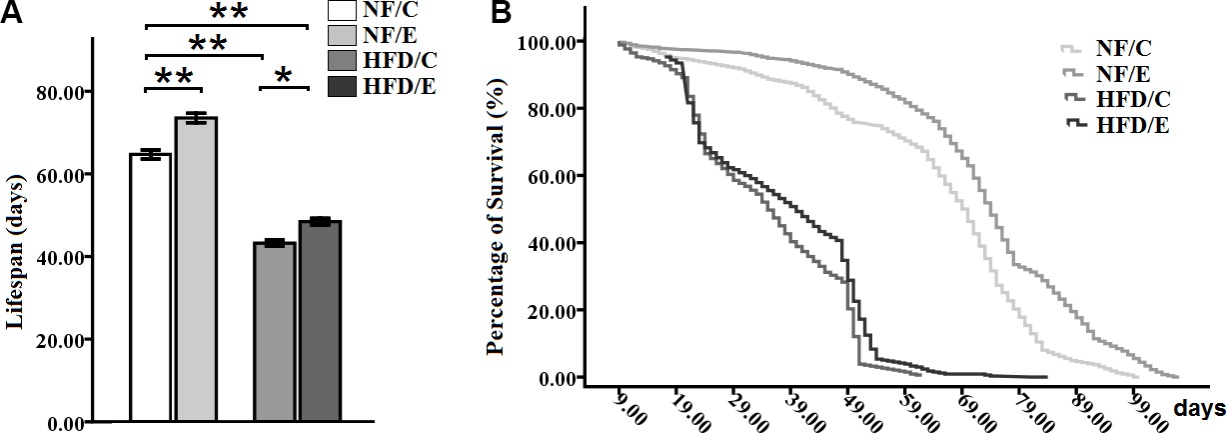
Effects of endurance training and HFD on lifespan in *Drosophila* (**A**) Average lifespan of flies cultured at four different groups. (**B**)Curves show the survival of flies. Results indicated that exercise could prolong lifespan, HFD could severely shorten lifespan, and exercise could protect flies from HFD-induced lifespan reduction. Using a non-parametric followed by a log-rank test among the NF/C, NF/E, HFD/C, and HFD/E group flies. Sample size of lifespan was 200 to 220 flies per group. Data are represented as means ± SEM. ^*^P < 0.05; ^**^P < 0.01.

Some studies confirmed that exercise did not only contribute to health span but also to lifespan, and that it delayed aging [35, 40]. Moreover, HFD-induced obesity leads to an unhealthy and shortened lifespan, and has created a heavy economic burden on human society [4, 5, 41]. Therefore, to determine whether long-term endurance exercise could extend HFD-induced decrease in lifespan, we examined the number of dead flies in every group. Our results showed that both exercise and HFD had significant effects on lifespan (2-factor ANOVA, *P* < 0.01, *P* < 0.01). The influences of exercise and HFD on fly lifespan were consistent with previous research. These two interventions had no interaction influence on the climbing index (2-factor ANOVA, *P* > 0.05). The average lifespan in NF/E group flies was longer than NF/C group flies (log-rank test, *P* < 0.01); the average lifespan in HFD/C group flies was shorter than NF/C group flies (log-rank test t, *P* < 0.01); the average lifespan in HFD/E group flies was shorter than NF/C group flies (log-rank test, *P* < 0.01); the average lifespan in HFD/E group flies was longer than HFD/C group flies (log-rank test, *P* < 0.01). These results suggested that exercise could effectively prolong the lifespan of flies, and protect flies from HFD-induced lifespan reduction (Figure 9A, 9B).

### Exercise increased HFD-induced and Age-related decrease dSir2 expression

Since the dSir2 gene was a key gene in regulating lipid metabolism and aging, few studies exploring whether exercise and HFD could affect the expression of the *dSir2* gene [42, 43]. We measured the dSir2 gene expression in fly bodies at different ages by qRT-PCR after exercise treatment and HFD treatment. Accumulating evidence suggests that NAD^+^/dSir2 not only play important roles not only in aging, but are also closely related to lipid metabolism and obesity. However, there is no immediate evidence that NAD^+^/dSir2 can regulate lipid metabolism and obesity, or that HFD-induced obesity can influence the expression of *dSir2.*

**Figure 10:**
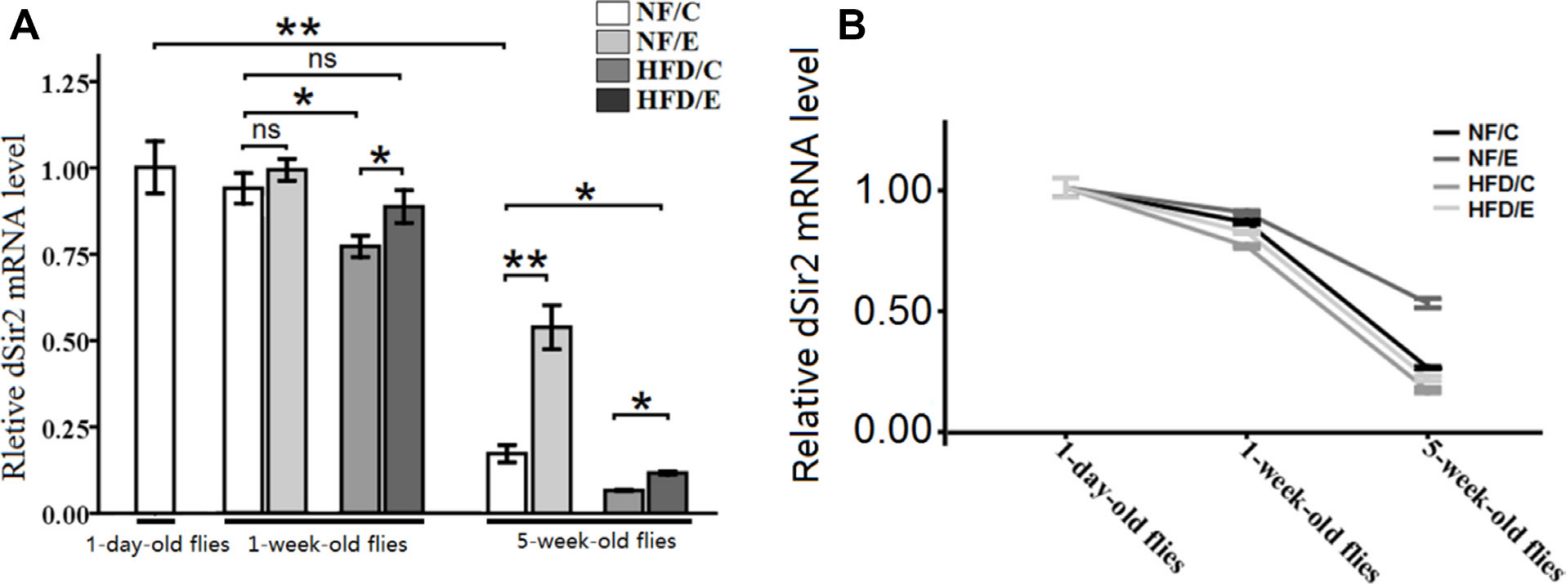
The expression of *dSir2* gene at different ages (**A**) The relative *dSir2* mRNA levels of whole fly bodies were assessed by qRT-PCR when flies were 1 day old, 1 week old, and 5 weeks old. (**B**) Exercise increased HFD-induced and age-related decrease *dSir2* expression. Independent-sample *t*-tests were used to assess differences between 1-day-old flies and 5-week-old flies. Using a one-way analysis of variance (ANOVA) followed by an LSD test among the NF/C, NF/E, HFD/C, and HFD/E group flies. Data are represented as means ± SEM.

In this study, we observed that exercise significantly increased the relative dSir2 mRNA levels (3-factor ANOVA, *P* < 0.01); HFD and age significantly reduced the relative dSir2 mRNA levels (3-factor ANOVA, *P* < 0.01, *P* < 0.01); exercise and HFD had no interaction influence on the relative dSir2 mRNA levels (3-factor ANOVA, *P* > 0.05); exercise and age had no interaction influence on the relative dSir2 mRNA levels (3-factor ANOVA, *P* > 0.05); HFD and age had no interaction influence on the relative dSir2 mRNA levels (3-factor ANOVA, *P* > 0.05); exercise, HFD, and age had no interaction influence on the relative dSir2 mRNA levels (3-factor ANOVA, *P* > 0.05). The relative dSir2 mRNA levels of 1-day-old flies were remarkably higher than that 5-week-old flies’ (independent-sample *t*-tests, *P* < 0.01), which suggested that the expression of *dSir2* declined with aging (Figure 10A).

In 1-week-old flies, the *dSir2* mRNA level of HFD/C group flies was significantly lower than of the NF/C group flies (LSD test, *P* < 0.05); The *dSir2* mRNA level of HFD/E group flies was significantly lower than of the HFD/C group flies (LSD test, *P* < 0.05). (Figure 9A)

At 5 weeks old, the *dSir2* mRNA level of NF/E group flies was significantly lower than of the NF/C group flies (LSD test, *P* < 0.01); The *dSir2* mRNA level of HFD/C group flies was significantly lower than of the NF/C group flies (LSD test, *P* < 0.05). The *dSir2* mRNA level of HFD/E group flies was significantly lower than of the NF/C group flies (LSD test, *P* < 0.01); the *dSir2* mRNA level of HFD/E group flies was significantly higher than of the HFD/C group flies (LSD test, *P* < 0.05). These results suggested that exercise could protect both young flies and old flies from HFD-induced decreased *dSir2* expression (Figure 10A and 10B).

## DISCUSSION

### HFD-induced obesity contributed to premature aging in Drosophila

Mobility, cardiac function, and lifespan are three important factors for measuring the degree of aging. Firstly, locomotor impairment with aging in flies results from the weakened resistance to oxidative stress [1]. Thus, HFD-induced obesity may result in earlier age-related locomotor impairment because of increased oxidative damage. In our study, we confirmed that both 1-week HFD and 5-week HFD could induce a fat accumulation and cutely reduce climbing capacity, which led to flies losing their climbing ability before becoming naturally too old to climb. Additionally, lipotoxic cardiomyopathy, a form of cardiac dysfunction, is caused by excessive lipid accumulation in myocardial cells [36, 37]. Recent studies reported that HFD- induced obesity, in *Drosophila*, could lead to excessive fat accumulation accompanied by severe heart defects, including increased frequency of arrhythmias, reduced cardiac output, increased non-contractile myocardial cells, and altered myofibrillar structure and collagen content [38, 39]. Similar to previous research, we also observed that both 1-week HFD and 5-week HFD could cause abnormal cardiac function, including increased heart rate. Besides, we also discovered that HFD could dramatically reduce fraction shortening. Both 1-week HFD and 5-week HFD distinctly increased fibrillation, which could increase the incidence of heart failure [38, 39]. Interestingly, some of these cardiac dysfunctions are similar to age-dependent deterioration of the heart, such as fibrillation [12]. These research results indicated that HFD-induced cardiac dysfunction may be a kind of premature aging of the heart. Finally, increasing evidence confirms that HFD can obviously decrease the average lifespan of flies [32, 44, 45], which is the most powerful and direct evidence suggesting that HFD-induced obesity accelerates aging and death. However, few studies have shown that HFD could affect the expression *dSir2* genes since the *dSir2* genes are closely related to aging [18–21]. In this study, we also observed that HFD acutely shortened fly lifespan. At the same time, we found that both HFD and aging would induce a decrease in *dSir2* expression, which indicate HFD may accelerate flies aging and death via down-regulate *dSir2* function, and this hypothesis requires more experimental evidence. Therefore, HFD-induced obesity accelerated aging in flies.

### Exercise protected Drosophila from the HFD-induced premature aging

Increasing evidence suggests that endurance exercise is a healthy and economical way to prevent and cure obesity, and is considered a good way to delay age-related functional decline [26, 34, 46]. In our study, we confirmed that exercise could protect Drosophila from the HFD-induced functional premature senescence. For example, exercise could effectively decrease fly TAG levels, which guarded against body fat accumulation and obesity [47]. During exercise, skeletal muscles participate in the metabolism of fat, which consumes TAG and supplies energy to the body. Besides, exercise can increase skeletal muscle mitochondria and strengthen muscle strength in both human and flies [11, 32, 34, 35]. Therefore, exercise training not only decreased lipid accumulation but also strengthened skeletal muscles performance, which led to an increase in mobility. Meanwhile, we found exercise could delay HFD-induced accelerated age-relate locomotor impairment. However, exercise did not obviously affected the climbing index in old HFD fly, and HFD was likely to accelerate flies age-related brain damage and muscle loss, which leads to the decreased negative geotaxis [44, 48, 49].

Furthermore, exercise may reduce fat accumulation in the heart since we found that exercise could protect fly hearts from HFD-induced cardiac dysfunction at different ages. One important factor was that when flies were doing exercise, their heart needed to spend a lot of energy for contraction, which may add to the consumption of fat to supply energy to the heart, and thus prevent lipid accumulation in the heart [12–14]. In our study, we also observed that long-term HFD contributed lipid droplets to heart in 5-week old flies, but long-term exercise could prevent its happen. Besides, from young to old flies, although HFD-induced lipotoxic cardiomyopathy could expedite age-related heart damage including heart rate and fibrillation abnormal increased, and fraction shortening acutely reduced, exercise could completely prevent this and maintain cardiac function at normal (NF/C group flies) state.

Finally, as is known, exercise training was good for lengthening health-span and reducing the risk of some diseases. In both humans and animals, numbers of studies have confirmed that moderate exercise improves the quality of life and prolongs lifespan [16, 27, 50]; however, obesity leads to a variety of diseases and a bad quality of life [4, 5]. This phenomenon confirmed again in our research. We also found that endurance exercise could up regulate *dSir2* expression and delay age-related *dSir2* expression decline in flies, and this might be one of several mechanisms through which endurance exercise extends longevity and health span. Increasing evidence has confirmed that moderate exercise does not only improve blood NAD^+^ levels but also muscle and cardiac NAD^+^ levels [51–53], which may result in eventual up regulation NAD^+^ activity in these tissues and organs to meet the demand of NAD^+^ metabolism during exercise. Several studies have reported that HFD induce a decrease in PGC-1 activity and FOXO activity in the fly heart, which leads to cardiac lipid accumulation and subsequent heart failure [23–25, 54]. Interestingly, dSir2 plays a pivotal role in PGC-1 function and FOXO function via NAD-dependent deacetylation [55–59], which indicates that dSir2 and NAD^+^ levels are associated with fat accumulation. Besides, a recent study has confirmed that Sir2 acts in the fat body to maintain insulin sensitivity and regulates metabolic gene expression such as *dHNF4* [60]. Since *dSir2* expression can be regulated by free NADH/NAD^+^ in cells, the *dSir2* expression and dSir2 activity were elevated indirectly by exercise [61]. However, this mechanism requires more research to be proved.

In conclusion, we show that endurance exercise improves climbing capacity, cardiac contraction, and *dSir2* expression, and it reduces body and heart triacylglycerol levels, heart fibrillation, and mortality in both HFD and aging flies. So, lifelong endurance exercise delays HFD-induced accelerated age-related locomotor impairment, cardiac dysfunction, death, and *dSir2* expression decline, and prevents HFD-induced premature aging in *Drosophila*.

## MATERIALS AND METHODS

### Fly stocks, diet and husbandry

The *w*^1118^ line was a gift from Xiu-shan Wu (Heart Development Center of Hunan Normal University). Normal food (NF) contained 10% yeast, 10% sucrose and 2% agar. The high-fat-diet (HFD) was made by mixing 30% coconut oil with the food in a weight to volume ratio with the NF [25].Virgin female *w*^1118^ flies were aged for 2 days after eclosion in tubes (25 flies/tube) containing NF and then transferred to NF or the HFD. We divided flies into the normal-food control group (NF/C), normal-food exercise group (NF/E), high-fat-diet control group (HFD/C), and high-fat-diet exercise group (HFD/E). Every group had 600 flies (24 tubes). Both HFD/C and HFD/E group flies were fed the HFD from 2 days old and were exposed to the HFD until all flies died. During the experimental time course, flies were housed in a 22 ± 1°C incubator with 50% humidity and a 12-hour light/dark cycle. This environment could keep the coconut oil food in solid state since the melting point of coconut oil is about 24°C, thus ensuring that flies would not get stuck in the oily food. Fresh food was provided every other day for the duration of the experiment. Identical groups of flies were either weighed or frozen at –80°C for determination of TAG content [25].

### Exercise training device and protocols

When constructing the exercise device, we took advantage of the flies’ natural negative geotaxis behavior to induce upward walking [26]. All exercise group flies started exercise from when they were 2 days old, and underwent a 5 week-long exercise program; we had observed that nearly all HFD group flies totally lost their negative geotaxis behavior by the age of 5 weeks. Vials, with the diet housing 25 flies each, were loaded horizontally into a steel tube that was rotated about its horizontal axis by an electric motor, with a gear regulating its shaft speed. NF/E group and HFD/E group severally had 24 vials training. Thus, with the accompanying rotating steel tube, each vial was rotated along its long axis, which made the flies climb. Most flies continued to respond by climbing throughout the exercise period. The few that failed to climb were actively walking at the inner wall of the vial [17, 27]. Flies were exercised in vials with a 2.8-cm inner diameter, rotated at 0.18 rev/s, 0.20 rev/s, 0.18 rev/s, 0.16 rev/s, and 0.14 rev/s. Flies were exercised for one and a half hours. Flies were exercised for 5 days, followed by a 2-day rest period, for a five-week period.

### Semi-intact Drosophila preparation and image analysis

30 flies were anesthetized with FlyNap for 2-3 min (a few flies were anesthetized with FlyNap 4-5 min since they were hard to narcotize), and the head, ventral thorax, and ventral abdominal cuticle were removed, exposing the heart and abdomen. Dissections were done under oxygenated artificial hemolymph. These semi-intact preparations were allowed to equilibrate with oxygenation for 15–20 min before filming. Image analysis of heart contractions was performed using high-speed videos of the preparations. Videos were taken 120–130 frames per second using a Hamamatsu (McBain Instruments, Chats worth, CA) EM-CCD digital camera on a Leica (McBain Instruments, Chatsworth, CA) DM LFSA microscope with a 10 immersionlens. To get a random sampling of heart function, a single 30-s recording was made for each fly. All images were acquired and contrast enhanced by using Simple PCI imaging software (Compix, Sewickley, PA). The heart physiology of the flies was assessed using a semi-automated optical heartbeat analysis program (gifted by Ocorr and Bodmer) that quantifies heart rate, fractional shortening, arrhythmia index, diastolic dysfunction, and fibrillation [28].

### Negative geotaxis assay

Flies were tested through climbing assay on the 1 day old, the last day of 1 week training, the last day of 3 week training, and the last day of 5 week training. The climbing apparatus consisted of an 18-cm-long vial with an inner diameter of 2.8 cm, and flies were allowed to adapt to the vial for ten minutes before assessing negative geotaxis. Sponges were placed in the ends of the tube to prevent escape while allowing air exchange [11]. With a light box behind the vials, the rack was tapped down five times and on the fifth, a timed digital camera snapped a picture after 8 seconds. The extent of climbing could be analyzed visually or by imaging software. Five pictures of each group were taken and averaged to arrive at a fixed score for each vial. Once photographs were collected, the number of flies in each of 9 equally-spaced quadrants per vial was charted. Flies were assigned individual scores based on which quadrant they reached within the allotted 8 seconds. Flies that reached the ninth and highest quadrant were given a score of 9, flies in the eighth quadrant were given a score of 8, flies in the seventh were given a score of 7 and so on. The total score for all the flies in a vial was tallied, and then divided by the number of flies in the vial to generate the “Climbing Index” for that trial. Each vial was subjected to 5 trials, and then the indexes from the five trials were averaged [27]. A total of 100–110 flies, 25 flies per tube, were measured for each group.

### qRT-PCR

To check the transcriptional expression of the *Nmnat* and *Sirt1*, 10 adult flies of each group were homogenized in Trizol. First, 10 μg of the total RNA was purified by organic solvent extraction from the Trizol (TRIzol, Invitrogen). The purified RNA was treated with DNase I (RNase-free, Roche) and used to produce oligo dT-primed cDNAs (SuperScript II RT, Invitrogen), which were then used as templates for quantitative real-time PCR. The rp49 gene was used as an internal reference for normalizing the quantity of total RNAs. Real-time PCR was performed with SYBR green using an ABI7300 Real time PCR Instrument (Applied Biosystems). Expression of the various genes was determined by the comparative CT method (ABI Prism 7700 Sequence Detection System User Bulletin #2, Applied Biosystems). Primer sequences of Sirt1/dSir2 were as follows: F: 5′-GCAGTGCC AGCCCAATAA-3′; R: 5′- AGCC GATCACGATCAGTAGA-3′. Primer sequences of Internal were as follows: F: 5′- CTAAGCTGTCGCACAAAT GG-3′; R: 5′-AACTTCTTGAATCCGGTGGG-3′.

### TAG measurement

For TAG assays, 8 flies were homogenized in phosphate-buffered saline (PBS) with 1% Triton-X and immediately incubated at 70°C for 10 min. Heat-treated homogenates were incubated with Free Glycerol Reagent (Sigma, St Louis, MO, USA) for 5 min at 37°C. Samples were assayed using a BioTek Synergy (Winooski, VT, USA) HT microplate spectrophotometer at 540 nm. TAG was determined by subtracting the amount of free glycerol in the PBS-treated sample from the total glycerol present in the sample treated with triglyceride reagent [29]. TAG measurement was repeated 5 times and required 40 flies in each group.

### Lifespan assays

Dead flies were recorded daily. Lifespan was estimated for each fly as the number of days alive from the day of eclosion to the day of death. Mean and median lifespan and survival curves were used to characterize the lifespan. Sample sizes were 200 to 210 flies per group [30].

### Statistical analyses

Independent-sample *t*-tests were used to assess differences between the 1-day-old control group and 5-week-old control group. A 3-way ANOVA was used to identify differences among the NF/C, NF/E, HFD/C, and HFD/E groups at different ages. 1-way analysis of variance (ANOVA) with least significant difference (LSD) tests was used to identify differences among the NF/C, NF/E, HFD/C, and HFD/E groups. Log-rank test was used to identify differences among the NF/C, NF/E, HFD/C, and HFD/E groups longevity. Analyses were performed using the Statistical Package for the Social Sciences (SPSS) version 16.0 for Windows (SPSS Inc., Chicago, USA), with statistical significance set at *P* < 0.05. Data are represented as means ± SEM.
